# Can Large-Scale Offshore Membrane Desalination Cost-Effectively and Ecologically Address Water Scarcity in the Middle East?

**DOI:** 10.3390/membranes12030323

**Published:** 2022-03-14

**Authors:** Daniel Janowitz, Sophie Groche, Süleyman Yüce, Thomas Melin, Thomas Wintgens

**Affiliations:** 1 STEP Consulting GmbH, Eupener Str. 30, 52066 Aachen, Germany; daniel.janowitz@rwth-aachen.de (D.J.); groche@stepconsulting.de (S.G.); yuece@stepconsulting.de (S.Y.); 2 Institute of Environmental Engineering, Rheinisch-Westfälische Technische Hochschule Aachen University, Mies-van-der-Rohe Straße 1, 52074 Aachen, Germany; 3 Aachener Verfahrenstechnik—Chemical Process Engineering, Rheinisch-Westfälische Technische Hochschule Aachen University, Forckenbeckstr. 51, 52074 Aachen, Germany; thomas.melin@rwth-aachen.de

**Keywords:** seawater reverse osmosis membranes, offshore desalination, large-scale desalination, water scarcity, artificial islands, renewable energy, cost-efficiency, carbon tax level, environmental impact, arid regions

## Abstract

The Middle East will face tremendous water scarcity by 2050, which can only be mitigated by large-scale reverse osmosis seawater desalination. However, the coastal land in the region is rare and costly, so outsourcing the desalination facility to artificial islands could become a realistic scenario. This study investigated the ecological and economic challenges and possible advantages of that water supply option by analysing conceptual alternatives for offshore membrane-based desalination plants of up to 600 MCM/y capacity. Key environmental impacts and mitigation strategies were identified, and a detailed economic analysis was conducted to compare the new approach to state-of-the-art. The economic analysis included calculating the cost of water production (WPC) and discussing the differences between offshore alternatives and a conventional onshore desalination plant. In addition, the study investigated the impact of a changing energy mix and potential carbon tax levels on the WPC until 2050. The results indicate that offshore desalination plants have ecological advantages compared to onshore desalination plants. Furthermore, the construction cost for the artificial islands has a much lower effect on the WPC than energy cost. In contrast, the impact of potential carbon tax levels on the WPC is significant. The specific construction cost ranges between 287 $/m^2^ and 1507 $/m^2^ depending on the artificial island type and distance to the shoreline, resulting in a WPC between 0.51 $/m^3^ and 0.59 $/m^3^. This work is the first to discuss the environmental and economic effects of locating large-scale seawater desalination plants on artificial islands.

## 1. Introduction

Water scarcity is of growing concern in many parts of the world, causing social and economic stress [1]. Arid and semi-arid regions will especially face tremendous shortcomings in water supply due to population growth and climate change. In Middle East countries such as Jordan and Palestine, the public and industrial freshwater deficits will likely be 1300 million cubic meters per year (MCM/y) in 2050, indicated recently by the SALAM initiative [2]. The SALAM initiative is a multi-lateral research project investigating innovative transboundary water transfer strategies between Israel, Jordan, and Palestine. In this respect, building large-scale seawater desalination plants at the Israelian coastline could be an essential part of the regional solution to produce and transfer the needed freshwater to the demand centres in Jordan and Palestine [3,4]. The water cost for desalination and especially water transport from the Mediterranean Sea to the main demand centre of Amman in the North of Jordan are much lower than Red Sea options [5].

Nowadays, reverse osmosis (RO) is the dominant technology for the realisation of large-scale seawater desalination plants due to its technical maturity and scalability [6]. The fundamentals of RO became common knowledge, and process innovations led to a significant decrease in energy demand from 20 kWh/m^3^ to 2.5–4 kWh/m^3^ [6,7,8,9]. Improvements in the efficiency of high-pressure pumps and energy recovery devices are still possible [9,10]. However, the required osmotic pressure limits the potential for further energy demand reduction. Therefore, desalination by reverse osmosis will always be an energy-intensive process [8]. In Israel, the existing desalination plants require a share of about 4 to 5% of the national energy demand. If fossil fuels are the basis for electricity production, the desalinated water has a very high carbon footprint between 0.4 and 6.7 kg_CO_2_eq_/m^3^ [11]. Israel plans to impose a carbon tax from 2023 to 2028, and so the cost of water production will likely increase if the energy mix remains fossil fuel-based [12]. Regarding climate change and the role that greenhouse gases play in it, future desalination plants will most likely be supplied with renewable energy [11]. In this respect, RO is the most appropriate technology for combining desalination with renewable energies since the energy demand is purely electrical [13].

However, environmental risks still exist concerning the intake and brine discharge of onshore desalination plants. Brine discharge can negatively impact marine ecosystems and water and sediment quality due to higher salinity and temperature, and process chemicals [14]. These risks amplify when the desalination capacity needs to be expanded at a specific location. Conventional seawater reverse osmosis desalination plants have a specific footprint from 0.16 m^2^/(m^3^/d) (Sorek I facility in Israel) to 0.20 m^2^/(m^3^/d), depending on the plant capacity [15,16]. There is potential to reduce the specific footprint further by vertical design and shared spaces in a modular build large-scale plant. An innovative design including a shared central administration, shared chemical and spare part storage, and shared pre-treatment facilities may lead to further reductions in specific footprint. Still, such a large-scale desalination plant competes with urban development for land use, especially in densely populated areas. In Israel, past extensive development made the open coast scarce leading to economic, social, and environmental conflicts [17]. Discussions and public protests have already led to a significant delay in realising an additional onshore desalination plant next to Acre [18]. Conflicts around appropriate land use, especially at the coastline, will likely increase in the future due to population growth, and as land value rises worldwide. This concerns land values in coastal cities such as Singapore, Hong Kong, Dubai, and Tel Aviv [19,20].

Recovering land with offshore structures such as artificial islands can be an approach to counteract these conflicts. Large projects show the feasibility of successfully constructing and maintaining artificial islands for decades. For example, the Kansai Airport in Osaka bay is located up to 7 km from the shoreline and has been in operation since 1994. Recently, Denmark has approved the ambitious plan to build an artificial island 80 km offshore as a hub for surrounding offshore wind farms. The first stage is scheduled to operate by 2033 [21]. Researchers discussed the economic and environmental aspects of building an artificial island off the Israeli coast almost 30 years ago [22], followed by a preliminary assessment in 2002 of constructing an airport offshore of Tel Aviv [23]. In addition, the Center for Urban and Regional Studies at the Technion published an Israel marine plan with a new vision and recommendations for policy measures for the future usage of Israel’s marine space in 2015 [24]. Recovering land for offshore seawater desalination with a capacity of up to 600 MCM/y, as it would be essential to mitigate a major part of the regional water deficit, is a new approach. So far, only desalination concepts implemented on ships, drilling rigs, or small-scale floats have been discussed [25,26].

This article assesses the ecological and economic challenges and possible advantages of large-scale desalination plants realised on artificial islands. The economic analysis includes calculating the cost of water production for discussing the differences between the offshore alternatives and a reference onshore desalination plant. Furthermore, the impact of changes in the energy mix and potential carbon tax levels on the water production cost is investigated until 2050. The study highlights future research opportunities and needs to enhance international discussions on implementing the new membrane-based offshore desalination approach.

## 2. Methods

### 2.1. Case Study and Artificial Island Design

**Figure 1:**
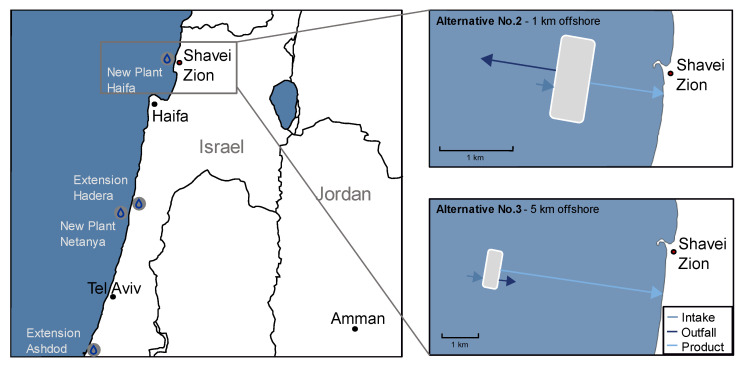
Map of the region including the considered areas for new desalination plants investigated in the SALAM initiative and a zoom-in on the study relevant alternatives No. 2 and No. 3 next to Shavei Zion.

Potential options for large-scale desalination plants were determined considering the most appropriate feed points into the existing water transport network and the proximity to the essential demand centres in Israel and Jordan [4]. From all options, the so-called “Haifa option” was investigated due to its proximity to the demand centre in Haifa and the possibility of constructing a tunnel as a connection to Lake Tiberias as natural water storage and distributor to Jordan. This article focuses on the conceptual alternatives for potential large-scale desalination plants on artificial islands in the north of Haifa. Figure 1 shows the regional map of the considered areas and an excerpt from the relevant study concepts. The size of the artificial islands and the distances to shore are drawn to scale. The required area for the concepts was determined to be 344.500 m^2^, resulting in a length of 1060 m and a width of 325 m.

Three offshore alternatives are conceptually developed at the study location next to Shavei Zion. The intake of the desalination plants is located at a minimum distance of 1 km from the artificial islands. The outfall is located at a distance of 2 km. These distances are based on the experiences of Israeli desalination plants [27]. Table 1 shows the relevant alternatives investigated in the economic and ecological analysis. The water depth was derived from bathymetry data in the Navionics [28].

The marine structure needs to ensure a stable base for protecting the investment in the large-scale seawater desalination plant for at least 50 years. Therefore, the detailed engineering design of marine structures includes the investigation of extreme design conditions (winds, waves, currents, tides), the geotechnical properties (soil mechanics and seismicity), the static design of structural elements, the environmental effects (water circulation, sediment transport) and construction and operational costs [29].

**Figure 2:**
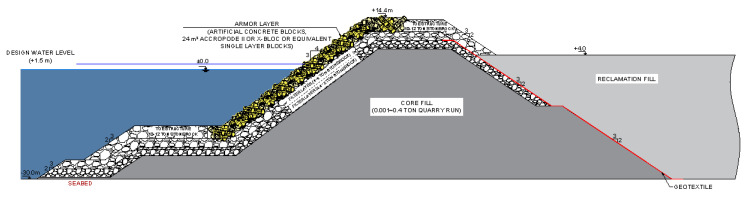
Conceptual cross-section design of alternative No. 3—5 km offshore −30 m water depth [33].

The artificial island’s conceptual design is based on collected and arranged raw wind data from NCEP (National Centers for Environmental Prediction) by the NOAA CFSR Global Model, including hourly average wind speeds and directions given in 1 h intervals for the period between 1994 and 2019 (227,905 lines of data) [30]. The associated significant wave heights were determined from wind speeds, fetch lengths, and storm durations using the simplified CERC Wave Prediction Nomogram (also known as Hassellmann Nomogram) for predicting waves in deep water according to the USACE-Shore Protecting Manual [31]. Given that the fetch lengths are high, the waves were calculated as duration limited. The extreme significant wave heights for different annual return periods and the generation of extreme value distributions were analysed by the Gumbel Distribution [32]. The design wave period has been taken as Ts = 13.0 s and Tm = 11.4 s for a characteristic wave steepness (height/length) of 0.04. The transformed significant wave height of the 100 year return period at a −30 m water depth contour was calculated by considering shoaling. The artificial islands were designed according to conventional rubble mound breakwaters. Rubble mound breakwaters are advantageous since their outer slopes force storm waves to dissipate energy effectively [29]. Additionally, necessary rocks are generally available, and local quarries can supply the stones. The water depth for the economically viable realisation of rubble mound breakwaters is limited to −30 m water depth [33]. Figure 2 shows the conceptual cross-section design of alternative No. 3—5 km offshore from the shoreline of Shavei Zion.

The artificial islands were conceptually designed using granular non-categorised material as reclamation fill protected and stabilised by an outer seawall (Figure 2) [33]. The outer seawall consists of a geotextile filter, core fill formed with quarry run, several filter layers, and an armour layer with concrete blocks. The armour layer protects the structure from erosion by currents and waves. Accropode II blocks were selected for the present study and were designed according to the CLI- Calculator based on the Hudson formula [34]. The filter layers underneath the armour layer prevent the core fill from washing out and protect the sub-layers from erosion. The first filter layer underneath the armour layer was selected according to the Accropode II Design Guideline [35]. According to standard filtering rules, the secondary filter layer was selected [29]. The crest elevation of the structure was determined according to wave run-up and overtopping calculations [29,31]. The artificial island should have a toe structure to ensure its stability by preventing scouring, undermining, and supporting against sliding. The geotextile between the core and reclamation fill has a filter and separation function that prevents washing out [29]. Seismicity and soil mechanics were not considered, so there may be a soil improvement requirement for these concepts, which may increase the investment cost. However, for a conceptual artificial island next to Tel Aviv, the seismic risk was stated to be low [36].

### 2.2. Desalination Project Context and Design

**Figure 3:**
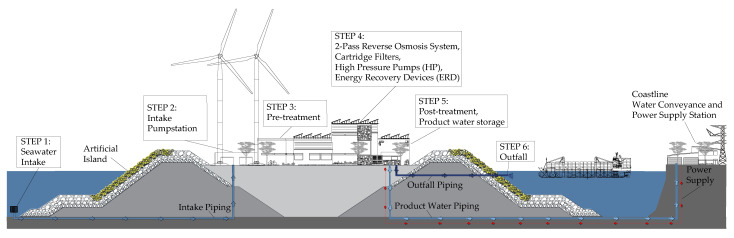
Desalination project context on the artificial islands.

After the conceptual design of the artificial island, the question of the context of the desalination plant arises. Facilities must be built on the artificial island for all important process steps needed in a conventional onshore desalination plant. Figure 3 shows the most important process components that were considered.

**Figure 4:**
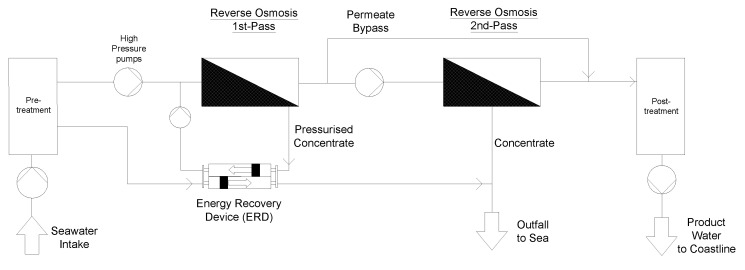
Simplified flow chart of two-pass reverse osmosis desalination with energy recovery.

The desalination plant is supplied with seawater via an intake screen system to minimise impingement and entrainment of marine organisms (STEP 1). Via intake piping, seawater is pumped through a second finer screen to the intake pump station (STEP 2). Within the pre-treatment, suspended solids, colloids, and organics are removed by clarification if necessary. However, the RO membranes only allow turbidity of <0.2 NTU and a silt density index (SDI) < 3–5 [6], which can be reached by ultrafiltration or multi-media filters within the pre-treatment (STEP 3). The reverse osmosis system was selected as a two-pass configuration in which the permeate from the first-pass is pressed through a second RO membrane pass (STEP 4). This is particularly important for boron removal using the product water as drinking water or agricultural water. The World Health Organisation recommends keeping the boron concentration in drinking water from desalination below 2.4 mg/L [37]. However, in agricultural water, the critical boron concentration varies with plant type, so values around 0.5 mg/L can already be critical [38,39]. In this respect, two pass systems showed their feasibility in effectively removing boron below the critical limits [6]. Figure 4 shows a simplified flow chart of the two-pass reverse osmosis system combined with energy recovery.

Cartridge filters serve as safety filters to prevent the membranes from being damaged by unseparated particles in the pre-treatment (Figure 3). Energy recovery devices are essential for reducing the total energy demand of the RO desalination process. Alkalinisation, remineralisation, pH adjustment, and disinfection are essential post-treatment steps to make the product water fulfill drinking water requirements (STEP 5). Concentrates and rinse water from the RO process and the pre-treatment are returned to the sea in an outfall system (STEP 6). In contrast to onshore desalination, the power supply must be provided via submarine cables and can be laid in parallel to the product water piping to the coast. All chemicals and additives, for example, antiscalants, must be brought to the island and stored there to optimise operations.

Private sector participation, including market-oriented tendering, is essential to reach competition, leading to fair-priced bids for the site-specific desalination project. Therefore, the membrane-based desalination plant was assumed to be procured according to the Build-Operate-Transfer scheme (BOT). BOT projects are essentially attractive for the private sector since governments provide take-or-pay guarantees reducing the commercial risk [40]. The engineering design, the permission, and the tendering of the artificial island can be expected to take two years, followed by a three-year construction phase. The design and tendering of the desalination plant could be carried out in parallel. However, the desalination plant can only be built after finalising the artificial island, so an additional 2–3 years should be expected for its construction. Therefore, the project could be completed in 2030, assuming immediate realisation. Time delays due to extensive permitting delays were excluded. The technical parameters for the study membrane-based desalination plant are shown in Table 2.

### 2.3. Method for Water Production Cost Calculation

The water tariff is the price off-takers as water supplies, or bulk users have to pay for desalinated water integrated with the country’s water supply network. Depending on the country, water tariffs are subsidised based on political will [40,42], so it is unsuitable as a technical assessment standard. Therefore we calculated the water production cost (WPC) excluding potential revenues of contractors’ subsidies.

**Figure 5:**
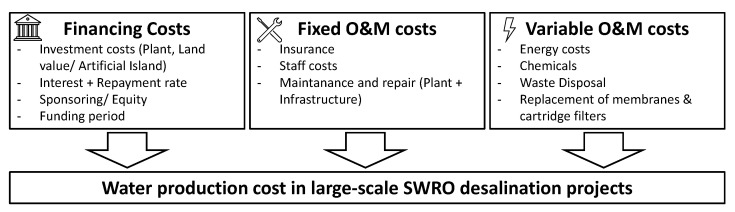
The main influences on the water production cost.

The WPC depends on the yearly fixed and variable costs for operation and maintenance and the financing costs (Figure 5).

The main difference in investment cost between onshore and offshore desalination is in the price for land acquisition compared to the construction cost and piping for the artificial islands. The project’s financing structure can include loans, equity, and potential sponsoring, resulting in the annuity essential in calculating the *WPC*. The *WPC* in $/m^3^ was calculated according to Equation (1) [43]:

(1) $$WPC=\frac{Annuity+O\&M_{fix}+O\&M_{var}}{Capacity}$$

whereas the *Annuity* [$/y] depends on the financing strategy according to Equation (2):

(2) $$Annuity=\left(Capex-Sponsoring\right)\frac{i\;{\left(1+i\right)}^{n}}{{\left(1+i\right)}^{n}-1}\;$$

Equation (2) includes the interest and repayment rate (*i* (%)) and the funding period (*n* (years)).

### 2.4. Cost Calculation Assumptions for the Desalination Project and Energy Mix

**Figure A1:**
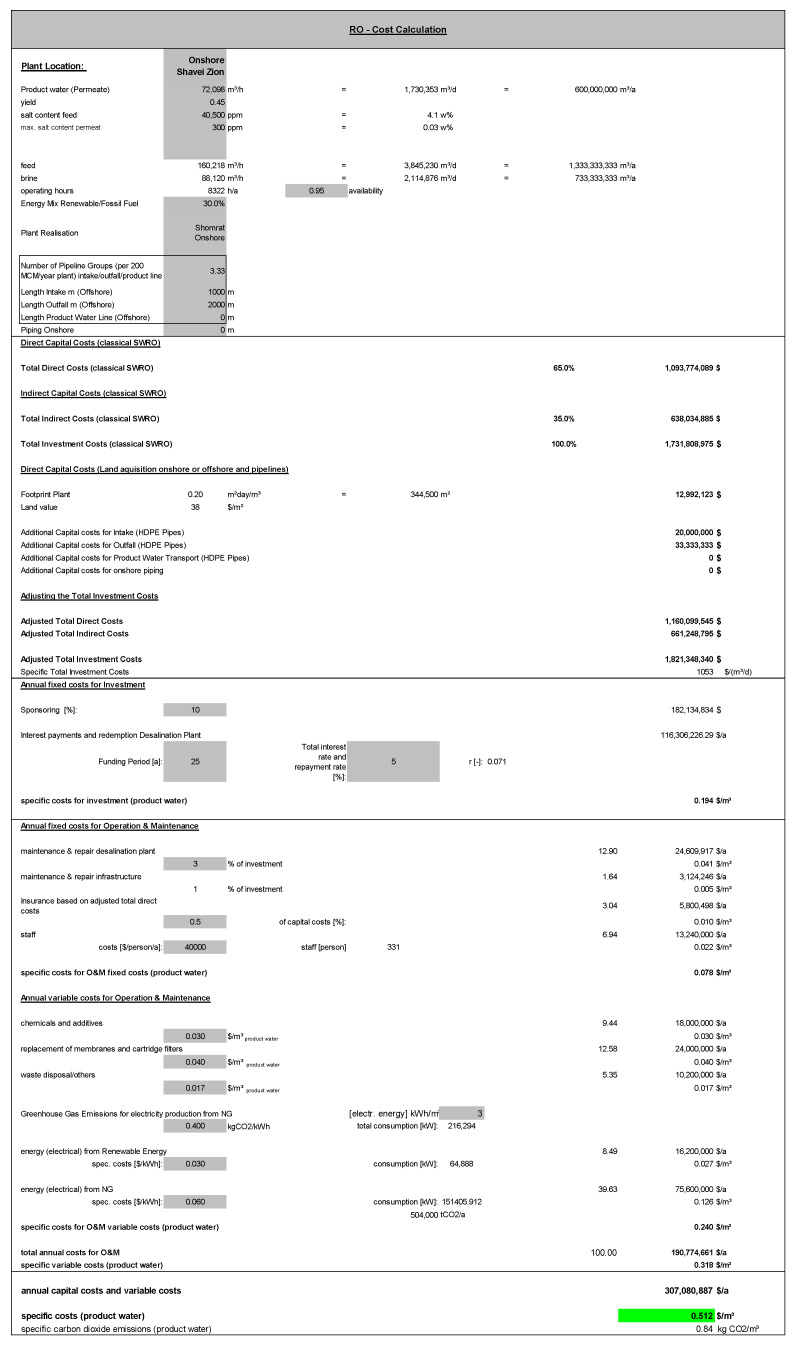
Detailed cost table for the economic analysis—onshore desalination.

**Figure A2:**
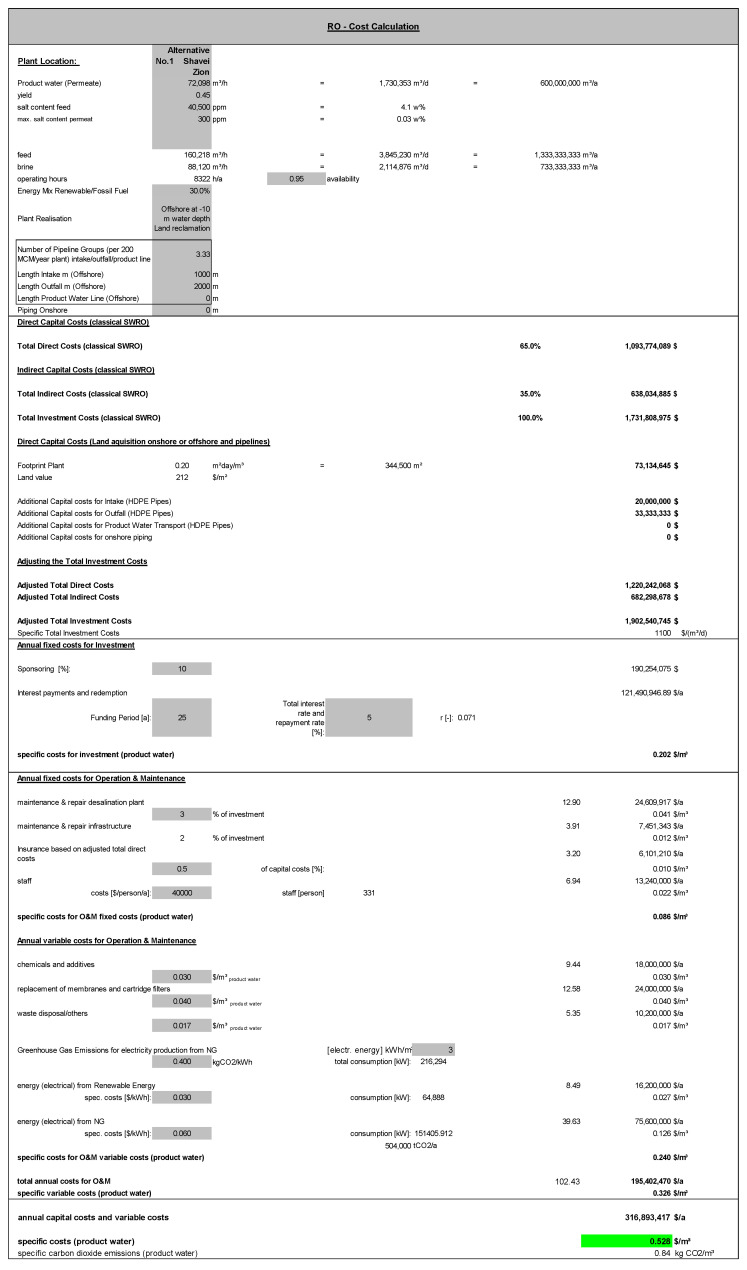
Detailed cost table for the economic analysis—Alternative No. 1.

**Figure A3:**
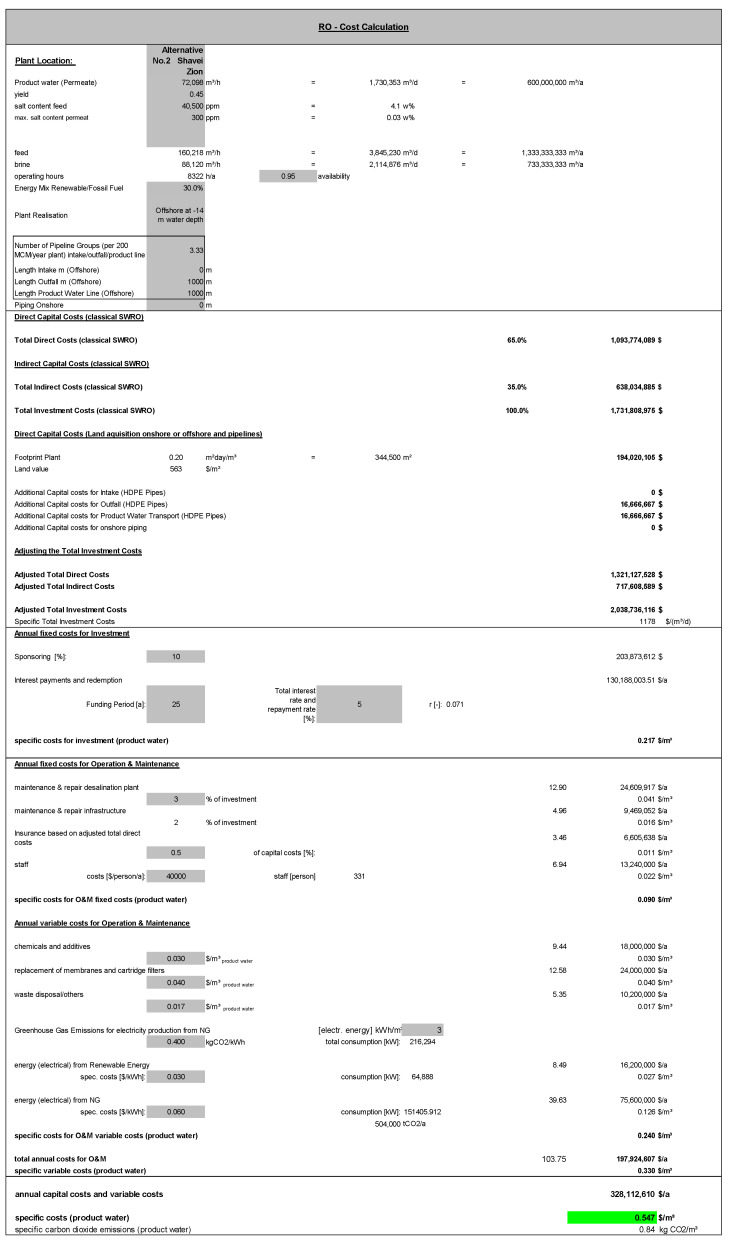
Detailed cost table for the economic analysis—Alternative No. 2.

**Figure A4:**
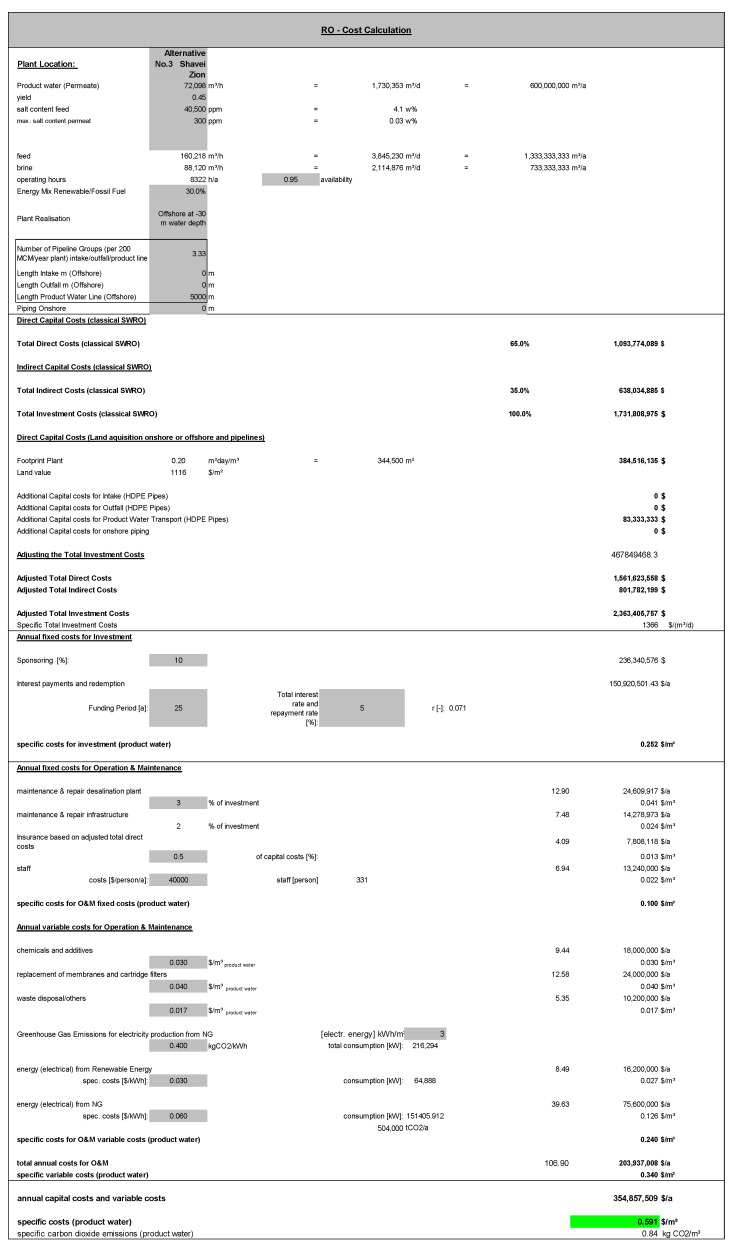
Detailed cost table for the economic analysis—Alternative No. 3.

Potential donors may provide equity, reducing the financial expenditures for the desalination project. For example, in the case of the Ashkelon desalination facility, 24% of the capital costs were sponsored [44]. Due to the anticipated high investment cost, we considered a share of sponsoring of 10% (Table 3). According to Israel’s typical BOT contract periods, the funding period was expected to be 25 years [32]. For reasons of comparability, the artificial island was assumed to be financed 25 years like the desalination plant. Nevertheless, the lifetime of the artificial island can be expected to be 50 years. The detailed cost tables showing the specific cost components are attached in Appendix A (Figure A1, Figure A2, Figure A3 and Figure A4).

The cost of energy makes up the largest part of the variable O&M costs. Therefore, it was essential to determine the future energy mix and the Levelized Cost of Energy (LCOE). The LCOE is the average generation cost, including CAPEX and OPEX at the point of connection to the grid [46]. The specific cost for electricity generation from photovoltaics (PV) significantly decreased and is already less than fossil-fueled sources today [47], so it was selected as the most economical renewable energy source to combine with desalination.

For simplification, we assumed an energy mix from photovoltaics (PV) as a renewable energy source and from natural gas-fueled Combined Cycle Power Plants (CCPP) (Table 4). According to the ministry of energy intentions, it can be expected that the share of renewable energy in the energy mix will likely increase to 30% in 2030 [48]. The 2050 targets have been tightened recently, so Israel aims to be carbon neutral by 2050 [49]. This political decision means that net-zero carbon emissions in the energy sector must be achieved. Therefore, the study’s target share in renewable energy has been set to 100% in 2050 (Table 4). Possible compensatory measures or carbon capture and storage measures were neglected.

The decrease in PV LCOE for 2040 and 2050 was based on the findings from Vartiainen et al. since the solar radiation in the study region is comparable [46]. However, the share of renewable energy can only be increased beyond ~30% grid penetration by energy storage systems [50]. Today, the commercial deployment of large-scale energy storage systems such as batteries or hydrogen with fuel cells is limited due to high costs, scarce material, environmental issues, and the necessity for performance and reliability improvements [51,52]. In addition, not every energy storage technology is generally applicable, considering all economic, social, and ecological conditions. For example, the geographic conditions must fit especially for the large-scale realisation of hydro pump storage systems. Therefore, it can be expected that a broad range of energy storage systems will be in the future energy mix [52]. One of the promising technologies is solar electricity storage in molten salt storage combined with a turbine block [50]. The reduced LCOE of PV can compensate for efficiency losses from thermal to electrical energy. Compared to batteries, this concept is ready for immediate implementation and is not limited by materials availability [50]. For simplification, we assumed that the share of renewable energy above 30% is supplied by the combination of PV with a mature energy storage technology. Cost reductions can be expected in the long term due to economy of scale and higher market penetration, so we assumed a slight decrease in costs over the decades until 2050 [50,52,53].

The LCOE of CCPP highly depends on the capacity and price for natural gas, which makes the LCOE impossible to predict for the next decades. In addition, we neglected the higher cost due to lower capacity factors in the case that flexibility in operation is necessary for renewable energy integration. Therefore, we set the CCPPs LCOE to a constant value of 0.06 $/kWh, which is in the lower range of the calculations from the International Energy Agency in 2020 [54]. Depending on the plant’s efficiency, the specific carbon dioxide emissions range between 360 and 575 g_CO_2_eq_/kWh_el_ for electricity generation. Upstream and downstream processes may also account for additional emissions of 60 to 130 g_CO_2_eq_/kWh_el_ [55]. Therefore, we assumed this study’s specific carbon dioxide emissions to be 400 g_CO_2_eq_/kWh_el_.

## 3. Results and Discussion

### 3.1. Assessment of the Offshore Construction Cost

Within the preliminary design phase, the construction costs of the artificial islands were estimated with quantities and unit price rates [29]. Table 5 shows the cost estimate for the artificial island at −30 m water depth using actual unit price rates. In addition, the estimations for Alternatives No. 1 and No. 2 are attached to Appendix B (Table A1 and Table A2).

The reclamation fill unit price was assumed to be for import material from quarries. If it is environmentally viable, most reclamation fill material can be gathered by dredging seabed material around the offshore location, which may dramatically reduce the unit price for reclamation fill. There is further potential in price reductions due to the large reclamation fill volume needed [33]. Investigations indicated suitable reclamation fill material at a distance between −30 and −50 m water depth 3–5 km from the coastline offshore of Tel Aviv [23]. Assuming a 50% decrease in the reclamation fill unit price could reduce the total cost estimation to around 420 Mio. $, resulting in a specific construction cost of 1219 $/m^2^. The quantities and unit prices have been conservatively estimated, and the actual local prices may vary and need to be specified in the following design stage. The detailed design phase includes investigating nearby quarries and possible dredging locations. If no nearby quarry with the desired material exists, far quarries are explored considering the logistics and transportation costs.

The same approach was used to determine the construction cost of alternatives No. 1 and No. 2 (Table A1 and Table A2 in Appendix B). Their construction costs are expected to be lower due to significantly reduced quantities in filling materials. In the case of alternative No. 1, the required reclamation fill is only one-third (3,393,600 m^3^) compared to alternative No. 3, due to the significantly lower necessary crest height of +8 m than +14.4 m in alternative No. 3 and the different water depths. In addition, fewer amounts of stones, rocks, and accropodes are necessary since the side facing the land does not need protective structures. The costs for the reclamation fill, stones, or rocks for the filter layer can be expected to be lower due to the direct land connection, reducing the unit prices.

But how do the estimated costs compare to similar projects? The results indicate that the specific construction cost of the artificial islands No. 1, No. 2, and No 3 are 287 $/m^2^, 760 $/m^2^, and 1507 $/m^2^, respectively (Table 6). Several land reclamation and artificial island projects are planned and constructed worldwide, but not every project fits the type rubble-mounted breakwater study design. For this reason, we have selected comparable projects and listed the most important benchmarks. The construction costs have been discounted to the current price level where necessary. Table 6 shows the data of comparable artificial islands projects.

The artificial islands of the Upper Zakum [56,57] and the Sateh Al Razboot [58,59] oilfields are comparably designed projects that have shown their feasibility for almost ten years. The water depths at Upper Zakum range between −6 m and −13 m, located 84 km offshore of Abu Dhabi. All Upper Zakum artificial islands combined have a total footprint of ~1.68 km^2^, five times the size of the artificial islands in the present study. Likewise, the North Sea Wind Power Hub is an innovative project that could be realised between 2030 and 2050 within the North Sea [60]. The specific construction costs are lower than the costs calculated in this study due to the almost 18 times larger surface area leading to cost advantages due to economies of scale. A large cost reduction factor can also be that dredging material is directly available at the target location for the reclamation fill.

However, the influences can be well explained by comparing alternative No. 3 (~1507 $/m^2^) and the conceptual airport offshore of Tel Aviv (~1395 $/m^2^). The reclamation fill levels of 4 m above sea level and crest heights with 14.4 m for alternative No. 3 and 16 m for the offshore airport are comparable [23]. The most significant differences are the water depth and the financing that increased 20 years ago the indirect cost to 45% due to high interest for the capital cost during construction. Reducing the indirect cost leads to an adjusted specific cost of 1263 $/m^2^ for the offshore airport’s island in 2022. This means that depending on water depth, the cost estimate of Alternatives No. 2 and No. 3 is comparable according to ±10% design accuracy.

In combination with the seawater desalination plant, additional costs must be taken into account for intake and outfall piping as well as pipelines for transporting the product water to the shoreline. Table 7 shows the resulting construction cost for all alternatives, the pipeline lengths, and the resulting specific costs for piping.

### 3.2. Assessment of the Water Production Cost of the Offshore Alternatives

**Figure 6:**
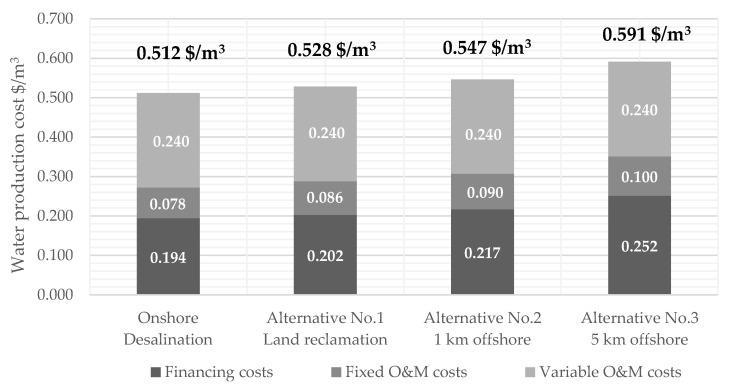
Comparison of the specific water production cost for all alternatives.

Large-scale desalination plants built on artificial islands compete with conventional onshore desalination plants. The results indicate that the water production cost (WPC) differs only slightly between the alternatives in 2030. Especially, the difference in the WPC between the onshore desalination plant and the most distant option from the shoreline (5 km offshore) is only ~0.08 $/m^3^ (Figure 6). The difference is ~48 Mio. $/y during the plant runtime for producing 600 MCM/y of freshwater mainly based on the annuities due to the artificial island construction cost, piping cost, and increased maintenance and repair expenditures.

The construction costs for the artificial island, so the WPC, directly correlate with the water depth due to the required filling material. The higher the water depth, the more filling material is necessary. Therefore, the difference in the cost for the filling material is ~140 Mio $ between alternative No. 2 and No. 3 due to the increase in water depth from −12 m to −30 m. Future projects need to consider the distance to the shore due to piping costs and the water depth.

In all alternatives, 64% (0.153 $/m^3^) of the variable O&M cost (0.240 $/m^3^) is on the cost of energy. Depending on the energy mix, additional carbon tax costs may arise in the future, increasing the WPC. Despite the increased capital cost due to the artificial islands and piping construction, the energy cost still has the most significant impact on the WPC. The result is in line with the energy cost of typical SWRO plants that are between 20–35% of the WPC [16].

In 2018, the WPC in seawater reverse osmosis plants ranged between 0.5 and 3 $/m^3^ (average 1.1 $/m^3^) depending on the capacity and local constraints as the feedwater salinity [16]. The tender for constructing the new Sorek II desalination plant with a capacity of 200 MCM/y even achieved a water price of 0.45 $/m^3^ in Israel [61]. Economics of scale, technical advancements in reducing the energy demand, competition in the tendering process, and favourable financing structure can be expected to have the most considerable influence on reaching such a low water price. Our results indicate that bringing the desalination plant to an offshore structure leads to a WPC in the range of today’s most economical plants. However, there are thresholds of the economy of scale for SWRO plants due to cost-effective sizes available on the market. The threshold for O&M costs is already reached at ~150–220 MCM/y capacity and for the SWRO investment costs for 73 MCM/y capacity [16]. Therefore, expanding the plant up to 600 MCM/year does not necessarily lead to a further reduction in investment costs.

Market-oriented tendering and procurement are essential to obtain offers for reasonably priced cost of water production. The WPC can only be kept low in case broader competition is reached. Therefore, it is advantageous to split the construction of the artificial island and the construction and operation of the desalination plant into two independent projects. This ensures that competition is achieved within the respective discipline. Build Operate Transfer (BOT) projects are especially advantageous for public utilities due to cost-effectiveness and precise transfer of risk to the public sector, including construction, technology, and operational risks [62,63]. However, the off-taker of the water purchasing agreements needs to ensure a continuous and reliable supply of electrical energy, connection to the water grid, and a solid land lease agreement [63]. For offshore desalination plants, this applies even more since the project is more complex.

### 3.3. Effect of a Carbon Tax on the Water Production Cost

**Figure 7:**
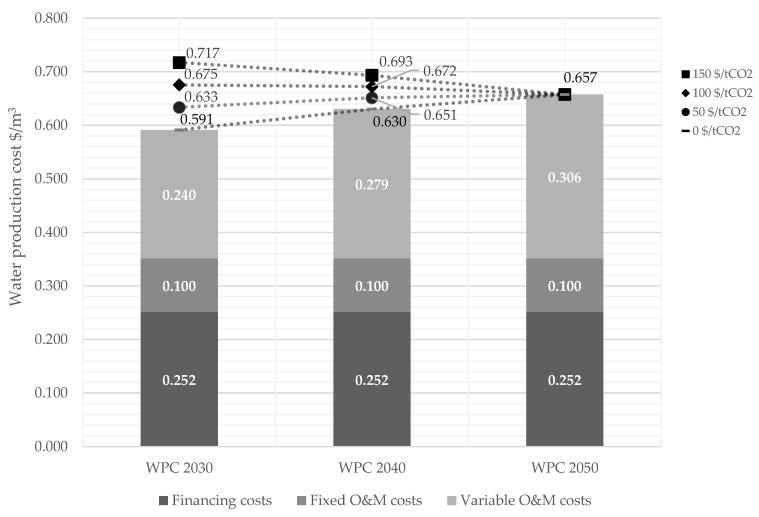
Comparison of water production cost varying the carbon tax level for Alternative No. 3.

Artificial islands are built for at least a 50 year lifetime. Therefore, their influence on the WPC must be evaluated in the context of governmental intentions to reduce carbon dioxide emissions significantly by 2050 [49]. Introducing a carbon tax can be a measure to achieve the essential carbon dioxide reductions, expecting carbon tax levels of up to 150 $/t_CO_2__ in 2050 [64]. The results indicate that the WPC strongly increases depending on the share of renewables and the level of a carbon tax (Figure 7). The strongest increase happens if renewable energy is as low as 30% without additional energy storage technology (WPC 2030). Carbon tax levels of 50–100–150 $/t_CO_2__ led to a rise in WPC of 7–14–21% compared to its absence.

This study assumed that energy storage technologies will increase the share in renewable energy to 65% in 2040 and 100% in 2050. In addition, we anticipated that global investments in renewable energies and storage would also lead to a slight reduction in their LCOE (Table 4). However, increasing the share of energy storage technologies in the energy mix in 2040 and 2050 will still lead to higher WPC than 2030. This leads to an optimisation potential of the energy mix from different sources for membrane-based desalination. Utilising wave energy and wind directly on the artificial island could reduce energy storage needs. Wave energy converters (WECs) could provide a reliable contribution to the supply, but there is still a need for research to reduce costs and bring the technologies to technical maturity [65,66].

Keeping the water tariffs predictable in the next decades will be important to ensure the planning security of companies and the population. Increasing the carbon tax level stepwise from 50–100–150 $/t_CO_2__ from 2030 to 2050 leads to low rises in WPC of 7–7–0% (Figure 7). Israel and Jordan recently signed a declaration of intent to transfer 200 MCM/y of freshwater from Israel to Jordan in exchange for 600 MW electrical energy from PV [49]. Ways must be found to ensure a fair trade of water and energy, regardless of a changing energy mix and carbon tax levels, as this study showed. In this respect, bartering water from Israel against renewable energy from Jordan could also be a beneficial approach to exclude carbon tax levels from the water-energy agreements [67].

### 3.4. Determination of Threshold Carbon Tax Level to Provide the Incentive to Couple Desalination with Renewable Energy

**Figure 8:**
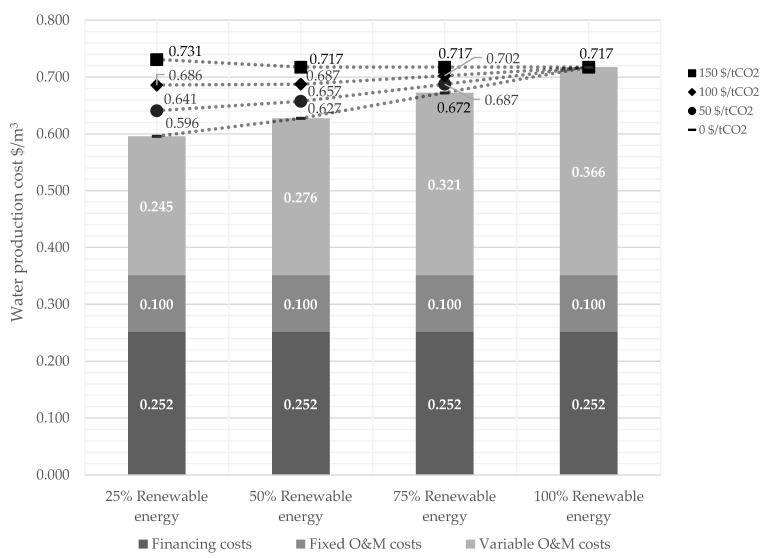
Comparison of the specific water production cost varying the share of renewable energy for alternative No. 3.

What would be the threshold carbon tax level necessary to push the complete switch to renewable energies for desalination? This question could be essential to answering if an Independent Power Producer (IPP) for renewable energy generation is included in the desalination project. The results indicate a significant rise in the WPC in case the share of renewable energy in the energy mix will already increase in 2030 (Figure 8).

Between 25% and 50% renewable energy shares, a sharp drop in WPC can be seen, especially at a carbon tax level of 150 $/t_CO_2__. A 25% increase in the renewable energy share reduces the WPC by 0.045 $/m^3^ due to the carbon tax savings. At the same time, the energy cost increases by only 0.031 $/m^2^, which explains the difference of 0.014 $/m^3^ in WPC.

For carbon tax levels of 50 and 100 $/t_CO_2__, there is no financial incentive to increase the share of renewable energy beyond 25%. However, for a threshold carbon tax level of 150 $/t_CO_2_,_ the lowest WPC is reached for a desalination plant entirely supplied by renewable energy, including storage. Based on the LCOE assumptions for 2030, the electricity generation cost of 0.093 $/kWh results in a WPC of 0.717 $/m^3^. Therefore, carbon tax levels would need to be higher than 150 $/t_CO_2__ to incentivise switching to a 100% renewable energy supply. This threshold carbon tax level decreases to ~127 $/t_CO_2__ in 2040 and 100 $/t_CO_2__ in 2050 due to the calculated decline in LCOE for PV and energy storage technologies.

### 3.5. Identification of Environmental Impact and Mitigation Strategies

The construction of an artificial island is a particularly severe interference with nature. Therefore, in planning a large-scale offshore desalination plant, mitigation strategies need to be developed to reduce environmental risks and increase social acceptance.

During the construction phase, the impacts directly correlate with the size of the marine structure and the water depth at the construction site. The mining of filling material, the construction of pipelines, and the artificial island itself can disturb valuable ecosystems resulting in a loss of biodiversity and habitat. The facts mentioned above are even more critical if the area is rich in biodiversity [36,68,69]. However, artificial islands can serve as new habitats and enable the development of unique and highly diverse ecosystems [68].

**Figure 9:**
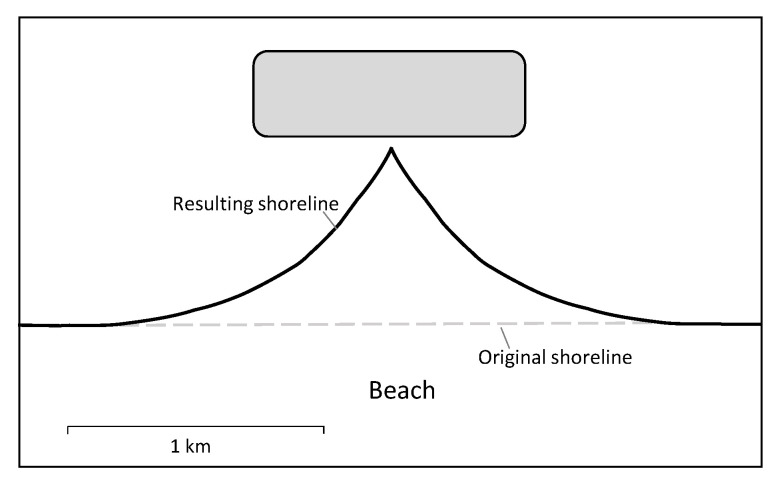
Expected beach profile of an offshore structure close to the coastline (extreme scenario) [33].

Artificial islands can change coastal circulation and sediment dynamics, depending on their length and distance to shore. As a result, beach erosion can occur [70]. However, the impacts can be mitigated with countermeasures such as sand bypassing and beach nourishment. The latter is the process of supplying the beach with the sand that has been lost due to erosion. This is only a temporary solution and needs to be repeated every couple of years depending on the extent of the erosion. Therefore, measures against erosion require repeated investments and must be included in the costs when planning an artificial island [71]. An example of a large-scale beach nourishment project is the “Sand Motor” in the Netherlands: a large amount of sand has been piled up on the coast, which is then distributed along the coast by currents, wind, and waves [72]. It is crucial to avoid hindering the longshore sediment transport since the Israeli coast is already prone to erosion due to a negative sand balance [36,73]. Therefore, possible impacts on the coastal environment should be minimised during an artificial island’s planning phase. An adapted size, shape, and distance to shore can significantly reduce the environmental impact [22,36,74]. The proposed artificial island does not affect coastal circulation or sediment dynamics if it is located further than 2.5 km from the shoreline [70]. Figure 9 shows the resulting beach profile of an offshore structure close to the coastline in an extreme scenario.

**Figure 10:**
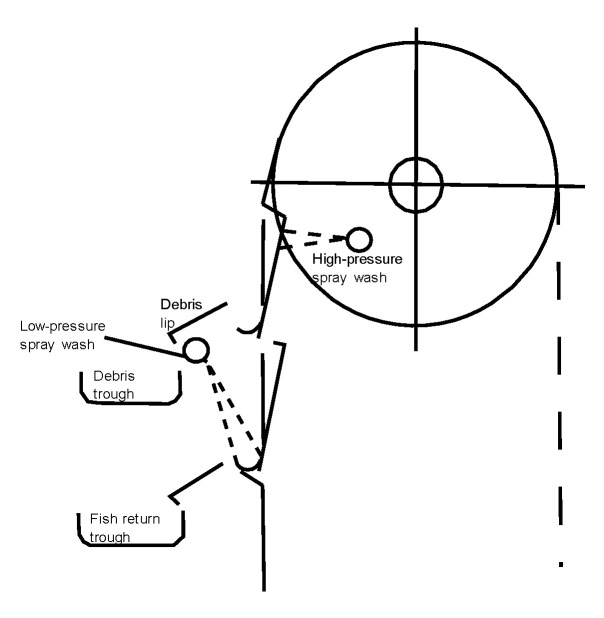
Fish return system adapted from [76].

The desalination process itself raises several environmental concerns. These include the intake of seawater, high energy consumption, and brine discharge. The seawater intake can adversely affect marine life through the impingement and entrainment of marine organisms, leading to biodiversity loss. Nevertheless, technical measures and innovations significantly reduce the impact. The reduction of intake velocity to 0.15 m/s reduces the impingement of fish and the risk of blocking [75]. In addition, innovative behavioural and mechanical barriers such as electric repel systems, filter screens, and fish return mechanisms should be implemented (Figure 10). 

These measures effectively prevent the harm of fishes larger than 8–10 cm [77]. However, the entrainment of smaller organisms such as juvenile fish, larvae, or eggs can only be minimised by locating the intake site in a less biologically sensitive area. Many desalination plant intakes are located in coastal and nearshore zones, even though these environments are usually biologically productive areas. Less biologically rich offshore zones start at around 20 m of water depth [75]. Additionally, reducing the entrainment of particulate and dissolved organic matter would improve the intake water quality. This would result in less fouling on the membranes and therefore reduce the pre-treatment and amount of chemicals needed [14].

Brine discharge also negatively impacts marine ecosystems, particularly the water and sediment quality, through increased salinity, higher temperature, and chemicals [14]. Due to the high salt content, brine has a higher density than water, sinks into low mixing zones at the bottom, and does not dilute quickly. This will, in particular, harm benthic organisms [75]. An active diffuser system can improve the dilution rate and discharge the brine at an inclined angle. However, choosing a suitable discharge location is still the best option to reduce the environmental impact. Habitats for rare and endangered species and regions with high biological productivity should be avoided [14]. Areas that are well-flushed and have low biological productivity are the least sensitive to the contents of the brine and are therefore a suitable discharge location. These environments are usually found in a greater depth and, therefore, further away from the shoreline [75].

Other emissions such as noise and light and their environmental impacts must also be considered. However, noise emissions from state-of-the-art desalination plants have a minor environmental impact [78]. As mentioned above, seawater desalination using reverse osmosis has a high energy demand, which will have to be covered by renewable energies in the future. Depending on the location of the artificial island, renewable energy sources could be utilised. Wind energy turbines, solar panels, and wave energy converters may be implemented on the island. Such options would allow synergies to be exploited [79].

### 3.6. Qualitative Assessment of the Environmental Impact of the Alternatives

**Figure 11:**
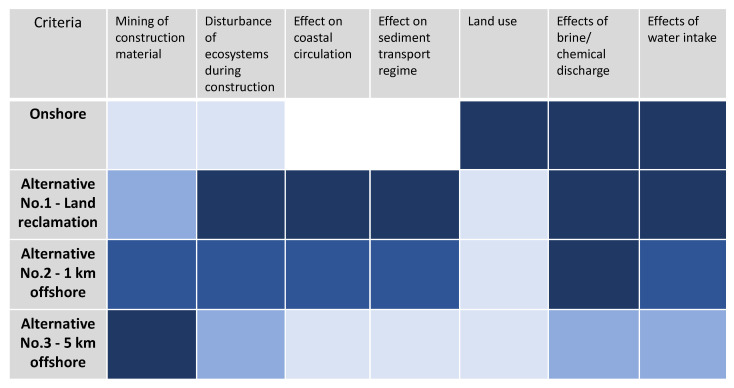
Heatmap for the environmental assessment of the alternatives.

Figure 11 shows the qualitative assessment of the alternatives and an onshore plant against criteria based on the identified environmental impacts. The alternatives were classified according to four colour gradations—the darker the box, the more severe the environmental impact. Blanked boxes indicate that there is no effect.

Table 1 is especially high due to the disturbance of valuable coastal ecosystems and significant changes in coastal circulation and sediment dynamics. Changes in coastal circulation and sediment transport are also expected under Alternative No. 2, while Alternative No. 3 has no impact on coastal circulation [70]. This makes it most likely that repeated investments for measures against beach erosion will be necessary for Alternative No.1 and Alternative No.2.

Outfall impacts are identical for the onshore plant, Alternatives No. 1 and No. 2, as they are located at the same site. In contrast, the outfall of Alternative No. 3 is located further offshore, where the environment is likely to be well flushed and less sensitive to brine discharge. Similarly, the intake of Alternative No. 3 is also located five kilometres offshore, resulting in reduced impingement and entrainment of organisms. The intake of the other three desalination plants is located one kilometre offshore, where the biodiversity is usually higher, making impingement and entrainment of marine organisms more likely.

The heatmap shows that the impact of the artificial island’s construction can be minimised by limiting the activity to offshore areas with low sensitivity and avoiding coastal areas. However, there has to be a trade-off between the environmental impact of intake and outfall, the effect of the artificial island on coastal circulation and sediment dynamics, and its increased need for construction material when going further offshore. The heat map indicates that this optimum is between 1 and 5 km offshore. However, in order to identify a suitable location, a local holistic environmental impact assessment needs to be compiled to identify the ecological consequences of the project [80,81].

### 3.7. Limitations of the Study

An environmental impact assessment (EIA) is a systematic process used to identify, evaluate and mitigate the environmental effects of a proposed project before major decisions and commitments are made [80]. A complete EIA is an essential step to build the basis for the realisation of artificial islands combined with a technical feasibility study. It was beyond the scope of the present study to quantitively weigh and evaluate the environmental impact of the offshore alternatives since site and location-specific data would be needed [14]. However, we qualitatively investigated the impact based on research findings to lay the groundwork for further research.

We neglected the influence of inflation, unforeseen events, and advancement in the RO desalination process on the WPC concerning the economic analysis. There may be significant advancements in reducing the energy demand or increasing the membranes lifetime. In addition, there may be advancements in cost-effective electrical energy storage in the field of batteries or fuel cells that could significantly enhance the combination of RO desalination with renewable energies. In this study, we selected technologies out of the development phase and available on the market today.

Within the framework of the present study, we intended only to carry out a preliminary design based on available data in the literature. The next step is to identify a specific site, including investigating nearby quarries and possible dredging locations. A pilot study should also take water samples at different water depths to assess the water quality. If the water quality is improved compared to conventional intake locations, the operating costs for the pre-treatment of the desalination plant could be reduced.

## 4. Conclusions

With increasing land value, permitting risks, and water demand, the pressure to recover useable land for desalination purposes will significantly increase in densely populated areas. To our knowledge, this work is the first to discuss the environmental and economic effects of locating large-scale seawater desalination plants on artificial islands. The ecological advantages concerning the intake and outfall of offshore desalination plants could exceed the risks, depending on the local conditions. In addition, artificial islands can serve as new habitats and enable the development of highly diverse ecosystems.

Economic of scale, technical advancements in reducing the energy demand, competition in the tendering process, and favourable financing structure can be expected to have the most considerable influence on lowering the water production cost (WPC) in the last decades. Outsourcing the desalination facility to an artificial island increases the WPC due to increased capital expenditures for piping and the structure itself. Nevertheless, our results indicate that still, the WPC is in the range of today’s most economical plants between 0.51 $/m^3^ and 0.59 $/m^3^. The investment costs for the artificial islands are highly dependent on the water depth at the construction site due to the required filling material. Depending on the study concept, the specific construction cost ranges between 287 $/m^2^ and 1507 $/m^2^. The distance to the shoreline determines the additional costs for undersea pipelines. However, this study reveals that the influence of energy cost on the WPC is still more significant than the financing cost for the additional construction costs for the artificial islands.

Due to the artificial islands’ lifetime of at least 50 years, the additional effect of potential carbon tax levels on the resulting WPC is not negligible. At this point lies a potential conflict between the intention to keep the WPC low and constant while steering the extension of renewable energy production and storage technologies. There may be advancements in cost-effective electrical energy storage in the field of batteries or fuel cells that could significantly enhance the combination of RO desalination with renewable energies on the way towards carbon neutrality. In the case of supplying freshwater from Israel to Jordan in exchange for renewable energy, as recently agreed in a joint declaration of intent, fair trade mechanisms should consider a changing energy mix for desalination and the level of a potential carbon tax.

The present study intends to motivate decision-makers to undertake further efforts for investigating the social acceptance and social-economic effects of large-scale offshore desalination plants on artificial islands. Decision-makers may also be inspired in identifying suitable locations for artificial islands and stimulate discussions on the implementation of on-site environmental and feasibility studies.

## Figures and Tables

**Table 1:** Alternatives for marine structures for large-scale desalination plants next to Shavei Zion.

Alternatives	Water Depth	Crest Height of the Structure above Chart Datum
No. 1—Artificial Island Land reclamation	−5 m	+8 m
No. 2—Artificial Island 1 km offshore	−12 m	+14 m
No. 3—Artificial Island 5 km offshore	−30 m	+14.4 m

**Table 2:** Technical parameters for the reverse osmosis desalination plant.

Parameter	Value	Unit
Type	BOT project Membrane-based Two-pass SWRO system	
Capacity	600	MCM/y
Availability	>95	%
Feed TDS	40,500	ppm [41]
Permeate TDS	300	ppm [41]
Recovery rate	~45	% [41]
Energy demand	3	kWh/m^3^ [16]

**Table 3:** Essential parameters for the economic assessment.

Parameter	Value	Unit
**Financing costs**		
Share of sponsoring/equity from total capital costs	10	%
BOT contract/funding period	25	years [45]
Sum interest rate and repayment rate	5	%
**Fixed O&M costs**		
Maintenance and repair desalination plant	3	%
Maintenance and repair infrastructure (onshore)	1	%
Maintenance and repair infrastructure (offshore)	2	%
**Variable O&M costs**		
Chemicals	0.03	$/m^3^ [6,16]
Replacement of membranes and cartridge filters	0.04	$/m^3^ [16]
Waste disposal	0.017	$/m^3^ [16]

**Table 4:** Variation in the energy mix and assumed Levelized Cost of Energy (LCOE).

Parameter	Value (2030)	Value (2040)	Value (2050)	Unit
Share of Renewable energy	30	65	100	%
LCOE PV	0.03 [50]	0.015 [46]	0.01 [46]	$/kWh
LCOE PV + Storage	0.12 [50]	0.11	0.10	$/kWh
Total LCOE Renewable	0.03	0.066	0.073	$/kWh
LCOE CCPP	0.06	0.06	0.06	$/kWh

**Table 5:** Cost estimate for the artificial island—Alternative No. 3.

Cost Item	Volume (m^3^)	Unit Price ($/m^3^)	Total Cost ($)
Reclamation fill	9,800,230	15	147,003,450
Core fill	7,652,648	15	114,789,720
Filter layer (0.4–2 ton)	953,964	35	33,388,740
Filter layer (4–6 ton)	796,019	35	27,860,665
Armour (24 m^2^ Accropode)	303,998	120	36,479,760
Toe structure (10–12 ton)	297,116	50	14,855,800
Back Armour (10–12 ton)	202,760	50	10,138,000
Direct investment cost	-	-	384,516,135
Indirect investment cost (Engineering, Permitting, Contingency)	35	%	134,580,647
**Total cost**			**519,096,782**

**Table 6:** Data of comparable artificial islands projects based on rubble mound breakwaters.

Artificial Island Projects	Construction Cost per Square Meter (2022)	Water Depth (Distance to Shore)	Project Description	Location
Alternative No. 1 (conceptual evaluation in this study)	~287 $/m^2^	−10 m (Land reclamation)	Rectangular Shape ~0.1 billion $, ~0.34 km^2^	North Haifa, Israel
Alternative No. 2 (conceptual evaluation in this study)	~760 $/m^2^	−12 m (1 km offshore)	Rectangular Shape ~0.26 billion $, ~0.34 km^2^	North Haifa, Israel
Alternative No. 3 (conceptual evaluation in this study)	~1507 $/m^2^	−30 m (5 km offshore)	Rectangular Shape ~0.52 billion $, ~0.34 km^2^	North Haifa, Israel
Upper Zakum Field Development (constructed) [56,57]	~469 $/m^2^ *^2^	−6 m to −13 m (84 km offshore)	Falcon-shaped, 2011–2014 ~ 0.63 billion $ (2011), ~1.68 km^2^	Abu Dhabi, UAE
Sateh Al Razboot oilfield (constructed) [58,59]	-	−13 m to −15 m (120 km offshore)	Falcon-shaped, 2011–2013 ~0.32 km^2^	Abu Dhabi, UAE
Artificial Island North Sea Wind Power Hub (planned) [60]	~333 $/m^2^	−18 m (100 km offshore)	Expected Realisation between 2030–2050 ~2 billion $, 6 km^2^	Middle of North Sea between Europe and the United Kingdom
Artificial Island for an airport offshore Tel Aviv (conceptual) [23]	~1395 $/m^2^ *^1^	~−19.5 m (1.35 km offshore)	Rectangular Shape ~2.1 billion $ (2002), 2.32 km^2^	Tel Aviv, Israel

*^1^ adjusted value using 55% inflation rate from 2002 to 2022. *^2^ adjusted value using 25% inflation rate from 2011 to 2022.

**Table 7:** Direct investment costs for all offshore alternatives including piping and the marine structures.

Alternative	Intake Piping *^1^	Outfall Piping *^1^	Product Piping *^1^	Piping ($) + Structure ($) = Total Cost ($)
No. 1—Artificial Island Land reclamation	1 km ~20 Mio. $	2 km ~33 Mio. $	Discharge already at shoreline	~53 Mio. $ + ~73 Mio. $ = 126 Mio. $
No. 2—Artificial Island 1 km offshore	Intake at site	1 km ~17 Mio. $	1 km ~17 Mio. $	~34 Mio. $ + ~194 Mio. $ = 228 Mio. $
No. 3—Artificial Island 5 km offshore	Intake at site	Outfall at site	5 km ~83 Mio. $	~83 Mio. $ + ~385 Mio. $ = 468 Mio. $

*^1^ The cost for the HDPE pipes was estimated concerning plant modules with a capacity of 200 MCM/y. For 400 MCM/y intake ~ 3 × 1800 mm OD pipes, for 200 MCM/y brine outfall ~ 2 × 1400 mm OD pipes, for 200 MCM/y product water transport ~ 2 × 1400 mm OD pipes.

## Data Availability

Data is contained within the article.

## Abbreviations

BOT	Build-Operate-Transfer
CAPEX	Capital expenditures
CCPP	Combined Cycle Power Plant
CERC	Coastal Engineering Research Center
CLI	Concrete Layer Innovation
EIA	Environmental Impact Assessment
ERD	Energy Recovery Device
HP	High pressure pumps
*i*	Interest and repayment rate
IPP	Independent Power Producer
LCOE	Levelized Cost of Energy
MCM/y	Million cubic meters per year
Mio.	Million
*n*	Funding period
NCEP	National Centers for Environmental Prediction
No.	Numbers indicating ordinal numeration
NTU	Nephelometric Turbidity Unit
OD	Outer diameter
O&M	Operation and Maintenance
O&Mfix	Fixed operation and maintenance costs
O&Mvar	Variable operation and maintenance costs
OPEX	Operational expenditures
ppm	Parts per million
PV	Photovoltaics
RO	Reverse Osmosis
SDI	Silt Density Index
SWRO	Seawater Reverse Osmosis
Tm	Mean wave period
Ts	Significant wave period
UAE	United Arab Emirates
USACE	United States Army Corps of Engineers
WEC	Wave Energy Converter
WPC	Water Production Cost

## Appendix B

**Table A1:** Cost estimate for land reclamation: Alternative No. 1.

Cost Component	Volume (m^3^)	Unit Price ($/m^3^)	Total Cost ($)
Reclamation fill	3,393,600	10	33,930,600
Core fill	1,074,222	10	10,742,220
Filter layer (0.4–2 ton)	263,391	20	5,267,820
Filter layer (4–6 ton)	202,652	20	4,053,040
Armour (18 m^3^ Accropode)	120,087	120	14,410,440
Toe (4–6 ton)	50,479	35	1,766,765
Back Armour (10–12 ton)	74,094	40	2,963,760
Direct investment cost	-	-	**73,134,645**
Indirect investment cost (Engineering, Permitting, Contingency)	35	%	**25,597,126**
**Total cost**			**98,731,771**

**Table A2:** Cost estimate for the artificial island: Alternative No. 2.

Cost Item	Volume (m^3^)	Unit Price ($/m^3^)	Total Cost ($)
Reclamation fill	5,421,158	15	81,317,370
Core fill	2,705,163	15	40,577,445
Filter layer (0.4–2 ton)	536,505	35s	18,777,675
Filter layer (4–6 ton)	382,045	35	13,371,575
Armour (18 m^3^ Accropode)	229,489	120	27,538,680
Toe (4–6 ton)	65,696	35	2,299,360
Back Armour (10–12 ton)	202,760	50	10,138,000
Direct investment cost	-	-	194,020,105
Indirect investment cost (Engineering, Permitting, Contingency)	35	%	67,907,037
**Total cost**			**261,927,142**
